# Protocol for a Retrospective Comparative Study to Determine the Effect of Two Different Biocomposite Suture Anchors on the Occurrence of Bony Ingrowth and Implant Reabsorption Following Arthroscopic Rotator Cuff Repair

**DOI:** 10.29337/ijsp.140

**Published:** 2021-07-29

**Authors:** Tanujan Thangarajah, Saho Tsuchiya, Ian K. Lo

**Affiliations:** 1 Department of Surgery, Section of Orthopaedic Surgery, University of Calgary, Calgary, Alberta, Canada

**Keywords:** Rotator cuff, Shoulder pain, Suture Anchor, Tendon repair

## Abstract

**Introduction:**

Surgical treatment of rotator cuff tears commonly entails reattachment of the ruptured tendon to its bony insertion using suture anchors. Suture anchor design has evolved from solid metal anchors to vented biocomposite anchors with potentially biologic consequences. Few studies have investigated the differences between different modern anchor design and materials, making it difficult to justify their use or cost.

**Objective:**

To compare the rate of bony ingrowth and implant resorption between a coil-type open-architecture biocomposite suture anchor and a vented screw-type biocomposite suture anchor, used for arthroscopic double-row rotator cuff repair.

**Methods and analysis:**

In this retrospective comparative study, a consecutive series of patients who undergo a double row rotator cuff repair using a coil-type open architecture biocomposite suture anchor in the medial row and a vented screw-type biocomposite suture anchor in the lateral row will be included. A sample size calculation demonstrated that 16 participants are required in each group. Primary outcome measures will be bony ingrowth and reabsorption of the suture anchor as measured on computed tomography (CT). Secondary outcomes will include patient reported outcome measures (The American Shoulder and Elbow Surgeons score and The Western Ontario Rotator Cuff questionnaire), range of motion, postoperative tendon integrity, and cyst formation.

**Highlights:**

- Open-architecture suture anchors facilitate the release of marrow constituents.
- The rate of bony ingrowth for coil- and screw-type anchors will be assessed.
- The difference in functional outcome between the two anchors will be assessed.

## 1. Introduction

### 1.1 Background

Suture anchors constitute the primary source of fixation during arthroscopic rotator cuff repair and have undergone several iterations as part of their development. Implant design and composition have evolved with corresponding advances in bioengineering. The primary goal of any such device is to produce a biomechanically stable construct that does not compromise healing of the tendon-bone interface and secondarily to minimize the short and long-term impact on the native anatomy. To achieve this various anchor design and biomaterials have been developed.

First-generation bioabsorbable suture anchors were commonly manufactured from polyglycolyic acid (PGA) leading to rapid absorption and an inflammatory reaction in the surrounding bone. As a result of this, tunnel widening and the collection of peri-implant fluid were reported [1]. To mitigate against these harmful processes, second-generation implants made from poly L-lactic acid (PLLA) were introduced. Whilst these led to slower biodegradation and fewer osseous reactions, they were associated with limited bony ingrowth. Biocomposite anchors were subsequently developed to specifically address this problem by incorporating osteoconductive materials such as beta-tricalcium phosphate (ß-TCP), hydroxyapatite and calcium sulphate [1]. These biocomposite materials have become increasingly popular in the attempt to achieve sufficient mechanical fixation to allow tendon healing to bone but in the long-term allow implant resorption and restoration of native bony architecture.

Material properties aside, suture anchors have additionally advanced from conventional solid screw designs to more ‘vented’ suture anchors. Vented suture anchors have additional holes in the otherwise solid anchor body which presumably allows bone in-growth through the implant and access of marrow elements to the bone-tendon healing interface. These vented suture anchor design have now evolved into coil-type open-architecture platforms, which theoretically allow even further bone ingrowth and marrow access despite a lower material load [2]. Despite their appearance, these anchors have shown similar biomechanical properties to traditional non-vented designs [3]. Understanding the *in vivo* behaviour of these suture anchors is crucial, but few studies have investigated the differences between suture anchors designs [2,4].

### 1.2 Objectives

To compare the rate of bony ingrowth and implant resorption between a coil-type open-architecture biocomposite suture anchor and a vented screw-type biocomposite suture anchor, used for arthroscopic double-row rotator cuff repair. We hypothesise that the coil-type biocomposite open-architecture design will lead to greater bone formation and enhanced anchor resorption.

### 1.3 Ethics statement

The study has been approved by the Conjoint Health Research Ethics Board (CHREB) at the University of Calgary.

## 2. Methods and Analysis

### 2.1 Study design

Retrospective observational study.

### 2.2 Setting

This retrospective study will be carried out in a tertiary referral centre for complex shoulder arthroscopy and involves patients that had surgery by the senior author (IKL) between April 2015 and February 2019.

### 2.3 Participants

A consecutive series of patients will be identified from their billing codes and their charts evaluated for implicit consent to take part in research. General eligibility will initially be confirmed by implicit written consent present in the medical records detailing the patient’s willingness to be contacted for involvement in a future study. For those patients, a member of the research team will contact them in order to gain formal consent for this particular study. An appointment for an in-person consultation will be issued so that the relevant outcome measures can be assessed.

Inclusion criteria for the study include:

1. A reparable partial/full-thickness supraspinatus/infraspinatus tear.
2. Surgery undertaken with a compression double-row technique exclusively using 4.75 or 5.5 mm Healicoil Regenesorb coil-type open-architecture suture anchors (Smith and Nephew, Ontario, Canada) composed of PLGA/B-TCP/Calcium suphate for the medial low, and 4.75 mm SwiveLock BioComposite vented suture anchors composed of PLLA/B-TCP (Arthrex, Naples, Florida, USA) for the lateral row.
3. One year minimum follow-up. Patient will be grouped according to length of follow-up to approximately 1 year follow-up and 2 years follow-up.

Exclusion criteria comprise the following:

1. A history of shoulder instability.
2. Any previous ipsilateral shoulder surgery.
3. Single row rotator cuff repair.
4. Partial reconstruction of the rotator cuff.

A sample size calculation assuming a type I error rate of 0.05, power of 80%, and standard deviation of 50 demonstrated that 16 participants will be required in each group.

### 2.4 Operative technique and postoperative rehabilitation

Preoperative rotator cuff tears will be diagnosed by ultrasonography or magnetic resonance imaging (MRI). The indication for surgery will be ongoing symptoms despite at least six months of non-operative treatment encompassing physiotherapy and analgesia. All operations will be performed by a single surgeon in the following manner:

– Patient will be operated under general anaesthesia with regional nerve block as per anaesthesia.
– The patient will be placed in the lateral decubitus position.
– A standard posterior viewing portal will be utilised for diagnostic arthroscopy with further portals being made according to the desired angle of approach required to reach the rotator cuff.
– Subacromial bursectomy will be carried out to aid visualisation and may involve an acromioplasty if an antero-lateral spur is identified.
– The rotator cuff tear will be debrided and repaired back to its footprint using a double row repair technique characterised by a 4.75 or 5.5 mm Healicoil Regenesorb anchor (Smith and Nephew, Ontario, Canada) for the medial row, and a 4.75 mm SwiveLock BioComposite anchor (Arthrex, Naples, Florida, USA) for the lateral row. In general one or two anchors are used medially and one or two anchors are used laterally.

A standardised postoperative rehabilitation protocol will be used. In brief, this entails a sling for six weeks, passive range of motion by six weeks, and active range of motion by eight weeks.

### 2.5 Outcome measures

Outcome measures will be assessed by a blinded musculoskeletal trained radiologist and will include the following:

– Primary – Bony ingrowth and reabsorption of the suture anchor.
– Secondary – Functional outcome, postoperative tendon integrity, cyst formation, and range of motion.

#### Radiological assessments

All radiographic measurements will be taken using a computer tomography (CT) scanner (Siemens, Medical Solutions, Germany) with 1.0 mm continuous slices through the shoulder. Inter-observer reliability will be determined by Cohen’s kappa coefficient.

A sterile suture anchor of each type being studied will be scanned by CT to obtain baseline data that will subsequently be used to determine whether the device has been completely reabsorbed and identify the nature of the material found in the location it previously occupied. A board-certified radiologist independent to the study and blinded to the intervention will use a semi-quantitative scoring system modified from that proposed by Haneveld et al to assess this at 12 months and 2-years postoperatively [5]:

– Grade 1 – Clearly visible structure.
– Grade 2 – Visible structure.
– Grade 3 – Partial visible structure.
– Grade 4 – Structure not visible.

Bony ingrowth into the anchor will be measured at 12 months and 2-years postoperatively. Oblique sagittal and coronal multiplanar reconstruction images will be reconstructed with short- and long-axis views oriented at the anchors along with axial images. Two orthopaedic surgeons blinded to the intervention will assess bony ingrowth using the ossification scale devised by Kim et al, and record the highest value from all image cuts taken (coronal, sagittal and axial) [4]:

– Stage 1 – Little or no ossification.
– Stage 2 – Some ossification but discontinuous or with a wide lucent rim.
– Stage 3 – Ossification with a thin lucent rim.
– Stage 4 – Good ossification and vague tract border.

Peri-implant cyst formation will be assessed on CT and defined as a gap where the hypodensity between the anchor and the bone exceed 1 mm in at least one image [4]. Ultrasonography will be used to evaluate postoperative tendon integrity.

#### Clinical outcomes

The Western Ontario Rotator Cuff questionnaire (WORC) and the American Shoulder and Elbow Society (ASES) scores will be used for functional outcome assessment at 12 months and 2-years following surgery [6]. The information will be recorded by the patients themselves on a secure online electronic database (RedCap) used by the study institution. Range of motion will be measured using a goniometer. The following indices will be recorded: forward flexion without scapula-thoracic involvement, external rotation at the side, external rotation at 90° abduction, and internal rotation using the highest vertebral level that the tip of the thumb could reach.

### 2.6 Data collection

Data will be collected from all adult patients, over the age of 16-years, undergoing double row arthroscopic rotator cuff repair by the principal investigator (IKL) using the technique described in section 2.4. The data fields will be based on existing information recorded for each patient as part of the local policy governing medical record keeping for patients undergoing surgery. Specific details that will be collected concern preoperative function of the shoulder being operated upon, operative data, and outcome data (***Tables 1*** and ***2***).

**Table 1:** Pre- and post-operative data.


GENDER, AGE	

Hand dominance	Left, right

Smoking	Yes, no

Diabetes	Yes, no

Occupation	High demand, low demand

Duration of symptoms before surgery	

Preoperative range of motion	Forward flexion Abduction External rotation

Postoperative range of motion	Forward flexion Abduction External rotation

Complications	

Primary outcome measure: – Bony ingrowth and reabsorption of the suture anchor	Measurement 1 Measurement 2 Measurement 3

Secondary outcome measures: – Tendon integrity – Suture anchor resorption (Haneveld classification) [5] – Pre- and post-operative ASES scores – Pre- and post-operative WORC scores	


**Table 2:** Operative data.


TYPE OF ANAESTHESIA	GENERAL, REGIONAL NERVE BLOCK ONLY, GENERAL ANAESTHESIA + REGIONAL NERVE BLOCK

Date/time operation started	

Date/time operation completed	

Side of operation	Left, right

Size of rotator cuff tear	

Tear retraction (cm/mm)	

Tendon quality	Good, thin, poor

Tendons involved	

Number of anchors medially (size, type)	

Number of anchors laterally (size, type)	

Concurrent shoulder pathology	

Long head of biceps	Tenotomy, tenodesis, other

Acromioplasty	

Other comments	


Data will be stored exclusively on an encrypted computer network that fulfils the clinical governance standards set-out by the local health authority. The system has been previously used to securely store data.

### 2.7 Methods for minimising bias

– Minimising selection bias – All patients under the care of the principal investigator will be screened for eligibility, and all those who meet the inclusion criteria will participate.
– Minimising reporting bias – By publishing this protocol with all stated outcome measures, selective reporting will be avoided.
– Outcome measures will be assessed by a professional blinded to the intervention.
– Patients themselves will complete their own functional outcome assessment using a online learning platform, after which the data will be securely stored.
– Conflicts of interests will be declared in their entirety.

### 2.8 Quality control

The quality of all submitted data will be checked by a dedicated independent research assistant. Specific indices that will be reviewed include the completeness of all data, the presence of outliers, and its adequacy. Missing data and results that cannot be interpreted will be reported, but the reasons for this will be examined and they will not be used in the final analysis.

### 2.9 Statistical methods

All statistical analyses will be performed on SPSS version 27.0 (IBM Corp., Armonk, USA). A Student t test will be used to determine differences between pre- and post-operative clinical outcome scores and range of motion. The Chi-squared test will be used to compare the rates of bony ingrowth, suture anchor reabsorption, cyst formation, and postoperative tendon integrity. The statistical significant level will be set at p < 0.05.

### 2.10 Statement of financial interests

IKL receives research support and royalties from, and performs consulting, for Smith & Nephew. The remaining authors (TT and ST) do not declare any conflicts of interests.

## 3. Discussion

Failure rates following rotator cuff repair have been reported to be as high as 46% [7]. This has been attributed to inadequate tendon-bone healing and so a number of biological strategies have been investigated in the literature. Suture anchors form the cornerstone of tendon reattachment to bone in this setting and their fixation is predicated upon the quality of the surrounding bone. Osteopenia has been identified as an independent risk factor for failure following rotator cuff repair and so increasing the bone mineral density at the site of repair would potentially mitigate against this [8,9].

Coil-type open-architecture suture anchors represent an exciting development because they are able to effectively liberate the bone marrow constituents integral to tissue healing [4]. Few studies have investigated their effect in the clinical setting, with none specifically evaluating bone formation [2,10]. This protocol was devised in order to compare the performance of this novel suture anchor to a commonly used vented anchor and so the results have the potential to influence the choice of device during surgery and improve the postoperative outcome. Strengths of this study include the homogenous patient population and the standardised surgical technique used throughout the cohort.

## 4. Ethics and Dissemination

The University of Calgary’s Research Ethics Committee has granted the principal investigator ethical approval for any retrospective clinical study that is conducted at the study institution. The study strictly complies with the regulations set out in that agreement and so no further approval will be required.

Results from the study will be presented at international scientific conferences focussed on arthroscopic surgery and published in peer-reviewed journals. The findings will additionally be disseminated to other surgeons across North America to guide policy.
